# Editorial: The use of technology in mental health occupational therapy

**DOI:** 10.3389/fpsyt.2026.1809565

**Published:** 2026-03-02

**Authors:** Bhing-Leet Tan, Tina Champagne

**Affiliations:** 1 Health and Social Sciences Cluster, Singapore Institute of Technology, Singapore, Singapore; 2 Occupational Therapy Department. Institute of Mental Health Singapore, Singapore, Singapore; 3 Cutchins Programs for Children and Families, Northampton, MA, United States

**Keywords:** artificial intelligence, extended reality (VR/AR/MR), mental health occupational therapy, occupational participation and engagement, technology - assistive/supportive, occupational therapy practice framework, digital tools

With the rapid adoption of technology in our everyday activities and in occupational therapy practice, it is now imperative for occupational therapy practitioners to investigate how we should harness technology appropriately and effectively in our assessments and interventions with people with mental health conditions. The American Occupational Therapy Association’s Occupational Therapy Practice Framework, 4th Edition (OTPF-4) identifies ‘products and technology’ as an environmental factor that plays an integral role in human occupations (2020, p. 37) (1). The OTPF also lists ‘virtual interventions’ as one of the interventions used to facilitate occupational engagement for health, well-being, and participation in life (2020, p. 62). While the use of social media tools and application software has been prevalent in mental health practice, more recent emerging technologies must be explored (2).

Vozza et al.’s scoping review highlighted that information and communication technology (ICT), such as social media and smart devices, could provide people living with psychotic disorders such as schizophrenia and schizoaffective disorders with tools to reduce social isolation, support peer connection, and empower self-management. However, occupational injustice surfaces when cost, digital literacy, cognitive difficulties and other psychiatric symptoms pose as barriers to access. A stark finding was the absence of any clinical intervention that directly addressed the use of ICT to facilitate occupational participation while mitigating the risks involved. Such risks include device loss, theft and excessive use of social media, which was reported to be associated with depression, anxiety, and sleep problems (3–6).

Occupational therapists use a variety of tools to assess performance skills, which are observable, goal-directed actions that include motor, process and social interaction skills (1). Lai et al.’s qualitative study explored the feasibility and acceptability of a semi-immersive virtual reality working memory task (VRWMT) for older adults. The authors found that the use of virtual reality-based cognitive screening was engaging and potentially more acceptable for users with higher technological familiarity. However, further research is needed to determine how data obtained from VR-based cognitive screening tools map to process skills and translate into occupational performance in daily living contexts. In the development and implementation of virtual reality modalities, it is essential for occupational therapy practitioners to adopt a user-centred design approach, incorporating personalized onboarding processes and simplified instructions to optimise user uptake and engagement.

Creative and therapeutic writing is one of the therapeutic expressive interventions that occupational therapy practitioners use to facilitate self-expression, reflection, acquisition of coping skills and identity development. Haertl’s perspective article explored the integration of digital writing tools into occupational therapy interventions. Writing tools such as blogs and Artificial Intelligence (AI)-powered chatbots have the potential to enhance clinical outcomes, provided that they are used with a clear clinical reasoning process within a sound theoretical framework. Ethical considerations are paramount, including privacy settings, content moderation and crisis planning, especially when using AI-powered or public platforms.

Not many occupational therapy practitioners are familiar with the term ‘digital twin’, which is a digital representation of a physical object, space, process etc. that enables visualization, monitoring, and prediction of real-world behavior (7). Through semi-structured interviews, Kaelin et al. identified four key aspects of participation in meaningful activities that could be represented by digital twins. Visual metaphors and simple graphics appeared to be useful in the provision of immediate feedback and longitudinal tracking.

Taken together, these findings suggest that occupational therapy practitioners should adopt a theoretically informed and intentional approach to leveraging technological tools in assessment and intervention, as part of the occupational therapy process. Technology affords opportunities for the delivery of virtual assessments and standardized scoring, thereby enhancing the consistency of instructions and improving the fidelity of standardized outcome measurements. Furthermore, the integration of technology expands the repertoire of therapeutic modalities available to occupational therapy practitioners, fostering the improvement of performance skills, performance patterns and occupational participation. Technologically mediated activities such as text-to-speech applications, the use of a variety of application software, digital image production, and navigation within virtual environments etc., are particularly relatable to young persons.

While technological applications are often used during individual sessions, purposeful integration into group-based interventions may facilitate social interaction of young adults with mental health conditions. However, technology can paradoxically function as an “excuse” for some service users to remain at home, particularly in the presence of psychiatric symptoms, avoidance of social engagement and avolition. As such, occupational therapy practitioners must carefully consider all variables involved and tailor the use of remote interventions to align with the occupational goals and recovery trajectory of each service user.

Occupational therapy practitioners have been harnessing technology (such as mobile applications, biofeedback etc) as preparatory or occupation-based interventions. What remains lacking is a robust, categorized list of the different types of technological interventions that may be used as part of occupational therapy practice, as well as research measuring their impact on occupational performance and participation. Therefore, the articles included in this Research Topic add to the body of knowledge by exploring some of the more recent technologies which can be used as part of occupational therapy practice. To advance knowledge and practice in this area, more research is needed to:

1. Develop and validate occupational therapy assessment tools that harness technology.
2. Explore and compile an extensive list of technological interventions that may be utilized as part of occupational therapy practice.
3. Investigate the effectiveness and efficacy of occupational therapy interventions that utilize digital tools, extended reality modalities, and artificial intelligence.
4. Understand the benefits and risks in the use of technology among service users with differing mental health conditions, strengths, needs and goals related to their everyday lives.
5. Define a framework that guides occupational therapists in the ethical and professional use of technology as part of occupational therapy practice.

As you explore the articles featured in this Research Topic, consider some of the following questions: How can occupational therapy practitioners facilitate persons with mental health-related needs access and make the most of technological tools to engage in meaningful everyday activities? What forms of support might be necessary to ensure the effective and consistent use of ICT as part of occupational therapy assessment and intervention? Lastly, recognizing that financial barriers may limit technology access for some individuals and populations, ongoing advocacy and support in acquiring appropriate technologies remains essential, to promote equitable and inclusive participation in community life.
